# Clinical Lectures on Diseases of the Eye

**Published:** 1865-01

**Authors:** E. L. Holmes

**Affiliations:** Chicago, Lecturer on Diseases of the Eye and Ear in Rush Medical College, and Surgeon of the Chicago Charitable Eye and Ear Infirmary


### CLINICAL LECTURES ON DISEASES OF THE EYE.

By E. L HOLMES, M. D., of Chicago,

*Lecturer on Diseases of the Eye and Ear in Rush Medical College, and Surgeon of the Chicago Charitable Eye and Ear Infirmary.*

### THE CHOROID—ANATOMY AND PHYSIOLOGY.

*Gentlemen*:—The study of this membrane, especially as affected by disease, has been rendered much more satisfactory and interesting by the discovery of the ophthalmoscope. Abnormal changes of the choroid, observed by the aid of this instrnment, during life, and further investigated by the microscope, are now much better understood than formerly. The classification of diseases is consequently more scientific and practical. I will endeavor to present to you the most important facts as now established.

The choroid is quite a thin membrane, having the retina on the inner, and the sclerotic on its outer surface. It is divided into two distinct portions. The first is a narrow band two or three lines wide, known as the ciliary body, and is situated in front of the anterior portion of the retina; the second, and by far the larger portion, is situated posterior to this border, and is called the true choroid. Throughout nearly the whole extent of this portion, it is quite loosely attached to the sclerotic and retina. It is firmly joined to the covering membrane of the optic nerve, where this nerve pierces it. It is also firmly attached to the retina at its anterior border or ora serrata. This latter attachment is so strong that the retina may be separated by serum from the choroid and yet hold firmly to the choroid along this border. The true choroid may be divided, according to some microscopists, into five distinct layers: 1st. A layer of pigment lying upon the inner surface of the sclerotic; 2d. A layer of larger vessels; 3d. The capillary layer; 4th. An extremely delicate transparent layer, and, 5th. The inner pigment layer. All these layers together are only 1-30 to 1-20 of a line in thickness.

The substance of the choroid is composed of the star and spindle shaped cells, with their anastomosing appendages, of which I have spoken, in describing the other tissues of the eye. Throughout the whole extent of the choroid, the pigment cells are quite abundant. The inner layer, upon which rests the retina, is almost wholly composed of this pigment.

The ciliary portion of the choroid is a narrow band, thicker at its anterior than at its posterior edge, lying between the anterior border of the retina and the periphery of the iris. Its general structure is similar to that of iris and true choroid, although it possesses less pigment. Its inner surface is corrugated into seventy or more folds, which are a continuation of the corresponding folds on the posterior surface of the iris. These are called the ciliary processes. Like the iris, this portion of the choroid contains two series of muscular fibres, the circular and the radiating, the relation of which to the function of accommodation of the eye to vision at different distances will be explained at a future time. At the ora serrata the true nervous structure of the retina ceases. The retina passes farther forward, however, in the form of a delicate, transparent, almost structureless membrane, which is quite firmly attached to the anterior edge of the true choroid and inner surface of the ciliary processes. As it approaches the periphery of the lens, it divides into two distinct layers, the inner one being attached to the posterior surface of the capsule of the lens, near its periphery, and the outer layer being attached to the anterior surface of the capsule. Between the layers and the periphery of the lens is the canal of Petit. This structure is the suspensory ligament of the lens, and is called the zonula of Zinn. Its function is intimately related to the accommodation of the eye to distinct vision, at different distances.

The choroid is principally supplied with blood by about twenty very minute branches of the ophthalmic artery—the posterior short ciliary arteries, which pass through the schrotic into the choroid near the optic nerve. These arteries supply also a small portion of blood to the ciliary processes and iris. The anterior ciliary arteries, which enter the globe a few lines posterior to the periphery of the cornea, give their blood principally to the ciliary portion of choroid and iris. The two long ciliary arteries enter the eye quite near the optic nerve and pass forward between the schrotic and choroid, one on each side of the globe, to the ciliary band and iris.

There are two series of veins in the choroid, one of which follows the general course of the ciliary arteries; the other is composed of five or six veins, which leave the eye half way between the optic nerve and cornea and at equal distance from each other. Each of these veins receives its blood from a series of smaller veins radiating around it like the spokes of a wheel, which are called the venæ vorticosæ.

The nerves of the choroid are the long and short ciliary nerves, composed of filliments from the third and fifth pair and from the sympathetic. These nerves pass through the substance of the choroid and are distributed almost wholly to the ciliary band and the iris. Some anatomists state that there are microscopic branches and nerve cells supplying the true choroid.

The pigment of the choroid, as also of the iris, is intended to absorb diffused light and thus render images upon the retina more distinct, as for the same purpose tubes of microscopes, telescopes, and the camera of the daguerreotypist are blackened upon their inner surface. Whenever this pigment is less abundant than it should be, as in the albino, vision is almost always indistinct, and the patient is dazzled by ordi nary light.

The iris, ciliary band and true choroid, sometimes called the sweal tract, may be considered as parts of one organ, the common function of which is the nutrition of the intraocular tissues. In addition to this common function, the iris, as we have seen, regulates the amount of light which enters the eye, and the ciliary bodies and zonula and zinn change the focus of the eye as it is fixed upon near or distant objects. The precise manner in which this process of nutrition is performed is not in all respects well understood. The aquious humor is undoubtedly the product of the iris and ciliary bodies. Diseases of the choroid often produce opacities of the lens and vitreous humor, and long continued inflammation of this coat of the eye is certain to terminate in atrophy of the globe. Diseases of the choroid, therefore, become of great importance to the student of ophthalmology.

An accurate diagnosis of these diseases often requires the assistance of the ophthalmoscope. For this reason you should bear in mind the normal appearances of the choroid as seen with this instrument. The rays of light reflected by the ophthalmoscope upon the transparent yet quite vascular ritina and the pigment layer of the choroid gives the characteristic yellowish red color of these tissues. The vessels of the choroid are usually covered by the pigment layer, and of course are not seen. In the negro, and individuals with dark skin and hair, the choroid contains more pigment, as seen by the darker shade of color revealed by the ophthalmoscope. In the albino, and individuals with light hair and skin, the color is much lighter, and there may be so little pigment that the choroidal vessels may be seen as broad, yellow lines, beautifully curved and looped together.

It should also be remembered that old age, as it removes the pigment matter from the hair and wrinkles the brow, often produces alterations in the ophthalmoscopic appearances of the choroid. The most frequent change is the absorption of pigment in irregular patches, which thus assume different shades of yellow brown. Not unfrequently in these cases, pigment is found in abnormal quantities scattered irregularly in small spots in different parts of the choroid.

